# Different processes shape the patterns of divergence in the nuclear and chloroplast genomes of a relict tree species in East Asia

**DOI:** 10.1002/ece3.6200

**Published:** 2020-03-24

**Authors:** Xiang‐Yu Tian, Jun‐Wei Ye, Tian‐Ming Wang, Lei Bao, Hong‐Fang Wang

**Affiliations:** ^1^ State Key Laboratory of Earth Surface Processes and Resource Ecology and Ministry of Education Key Laboratory for Biodiversity Science and Ecological Engineering College of Life Sciences Beijing Normal University Beijing China; ^2^ Germplasm Bank of Wild Species in Southwest China Kunming Institute of Botany Chinese Academy of Sciences Kunming China

**Keywords:** East Asia, genetic marker, isolation by distance, isolation by environment, isolation by instability, regional history

## Abstract

Isolation by spatial distance (IBD), environment (IBE), and historical climatic instability (IBI) are three common processes assessed in phylogeographic and/or landscape genetic studies. However, the relative contributions of these three processes with respect to spatial genetic patterns have seldom been compared. Moreover, whether the relative contribution differs in different regions or when assessed using different genetic markers has rarely been reported. *Lindera obtusiloba* has been found to have two sister genetic clades of chloroplast (cpDNA) and nuclear microsatellite (nSSR), both of which show discontinuous distribution in northern and southern East Asia. In this study, we used the Mantel test and multiple matrix regression with randomization (MMRR) to determine the relative contributions of IBD, IBE, and IBI with respect to *L. obtusiloba* populations. Independent Mantel tests and MMRR calculations were conducted for two genetic data sets (cpDNA and nSSR) and for different regions (the overall species range, and northern and southern subregions of the range). We found a significant IBI pattern in nSSR divergence for all assessed regions, whereas no clear IBI pattern was detected with respect to cpDNA. In contrast, significant (or marginal) divergent IBD patterns were detected for cpDNA in all regions, whereas although a significant IBE was apparent with respect to the overall range, the effect was not detected in the two subregions. The differences identified in nSSR and cpDNA population divergence may be related to differences in the heredity and ploidy of the markers. Compared with the southern region, the northern region showed less significant correlation patterns, which may be related to the shorter population history and restricted population range. The findings of this study serve to illustrate that comparing between markers or regions can contribute to gaining a better understanding the population histories of different genomes or within different regions of a species' range.

## Graphical abstract

We infer that different markers reflecting divergent processes may owe to the heredity and ploidy difference of genomes, while divergent extent of significant patterns in northern and southern regions may owe to different population history between regions.

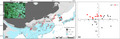

## 1 BACKGROUND

Disentangling the various contributions of geographical, environmental, and historical climatic dynamics to population genetic structures could provide significant insights into the species diversification (Lee & Mitchell‐olds, 2011; Meirmans, 2012; Wang & Bradburd, 2014; Wright, 1943). Under the isolation‐by‐distance (IBD) scenario, gene flow is considered to be negatively correlated with geographical distance, and accordingly genetic differentiation between populations is expected to be positively correlated with distance (Hedrick, 2005; Meirmans, 2012; Wright, 1943). In contrast, the isolation‐by‐environment (IBE) hypothesis proposed that it is differences in environmental variables between sites that can explain most of the observed variance in gene flow and implies that local adaptation may occur and restrict the gene flow between sites (Manthey & Moyle, 2015; Sexton, Hangartner, & Hoffmann, 2014; Wang & Bradburd, 2014). In addition to IBD and IBE, historical climatic fluctuations, particularly glacial–interglacial cycles during the Pleistocene era (2.58–0.0117 million years ago, hereafter, Ma), have markedly influenced species distribution and demographic history (Qiu, Fu, & Comes, 2011; Soltis, Morris, McLachlan, Manos, & Soltis, 2006). These fluctuations in species distribution may subsequently lead to an instability in habitat connectivity and thereby contribute to enhancing population genetic differentiation. Isolation by instability (IBI) denotes a negative correlation between the extent of the historical instability of habitat connectivity and gene flow. Although both IBE and IBI can be calculated based on environment data, these processes differ in important respects, with IBE being characterized by the influence of differences in habitat environment on gene flow, whereas IBI is characterized by the influence of long‐term habitat instability due to climate change and its resistance on dispersal between habitats. Previously, a number of studies have compared IBD and IBE to evaluate the contribution of local adaptation to present‐day patterns in spatial genetic (Pelletier & Carstens, 2018; Sexton et al., 2014; Shafer & Wolf, 2013; Wang, Glor, & Losos, 2013). However, although the effect of historical climatic fluctuations on genetic diversity has been frequently described in phylogeographic studies, IBI has seldom been examined and compared with IBD and IBE [for rare examples, see *Lerista lineopunctulata* Duméril & Bibron, 1839 (Sexton et al., 2014), *Quercus lobata* Née (Gugger, Ikegami, & Sork, 2013) *Hypsiboas lundii* Burmeister, 1856 (Vasconcellos et al., 2019)].

**FIGURE 1:**
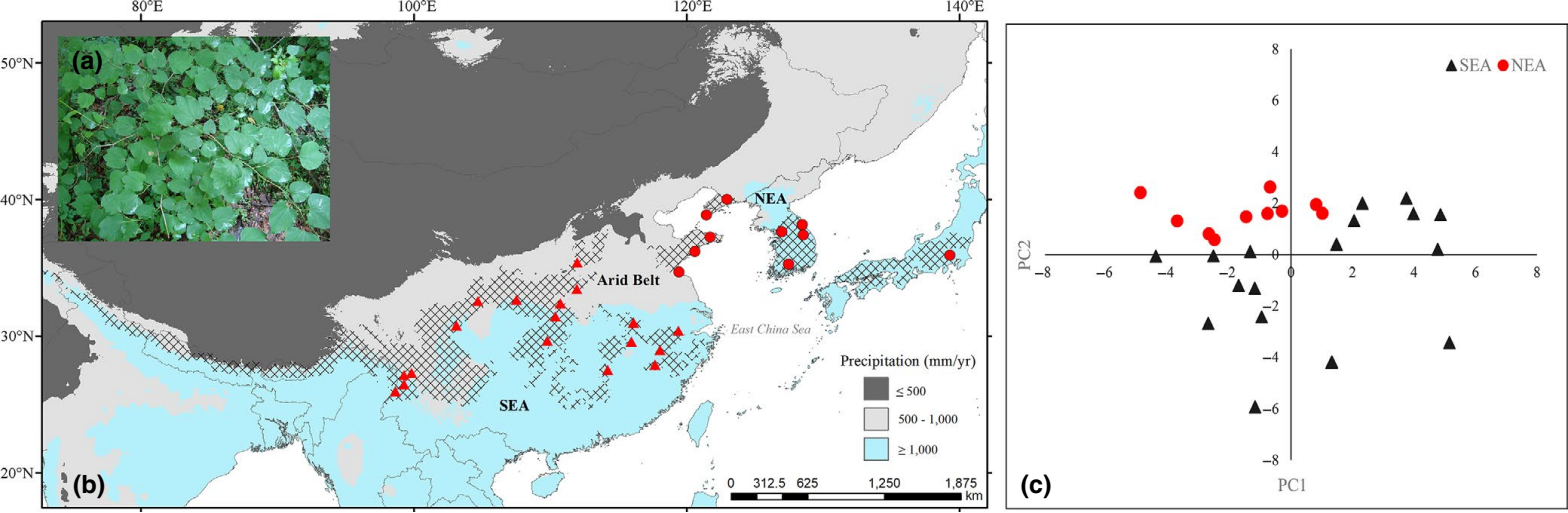
(a) An individual plant of *Lindera obtusiloba* showing the typical trilobed leaves. (b) The species range of *L. obtusiloba* (hatched area) and locations of the populations sampled in this study. The two sister genetic clades reported in a previous study (Ye et al., 2017) are denoted by different symbols: NEA clades, red circle; SEA clades, red triangle. The different colored areas of the map indicate the distribution of annual precipitation. This indicates that the approximate range of the two Cenozoic relict flora refugial regions in East Asia (NEA and SEA) is characterized by annual precipitation greater than 1,000 mm (Milne & Abbott, 2002). The region of low precipitation dividing the NEA and SEA (precipitation <1,000 mm) is referred to as the “arid belt,” which is suggested to constitute a climatic barrier for species migration between the NEA and SEA regions (Milne & Abbott, 2002). (c) The PCA results for all sampled populations, indicating bioclimatic differences between populations. The black triangles and red circles represent SEA and NEA clade populations, respectively

Owing to its extensive physiographical heterogeneity, in conjunction with frequent historical changes in climate and sea level, East Asia currently harbors the world's highest diversity of temperate flora (Qian & Ricklefs, 2000). It has been suggested that Cenozoic (66–23 Ma) relict genera in East Asia can be subdivided into two refugial regions (Milne & Abbott, 2002), namely the northern refugial region (NEA), comprising Japan, Korea, and adjacent areas of Northeast China, and the southern region (SEA), comprising south and southeastern China with extensions into Himalayan areas. These two refugial regions are divided by a low‐precipitation zone, referred to as the “aridity belt” (Figure 1), which developed during the Eocene (56–33.9 Ma) and has persisted to the present day (Chen, Deng, Zhou, & Sun, 2018; Donoghue, Bell, & Li, 2001; Milne & Abbott, 2002). The arid belt is believed to represent a climatic barrier that restricts gene flow between the NEA and SEA regions (Bai, Wang, & Zhang, 2016; Milne & Abbott, 2002; Ye et al., 2017). Although studies over the past two decades have begun to focus on the spatial genetic patterns of temperate plant species in both the NEA and SEA regions and their responses to historical climate change (Chen et al., 2018; Harrison, Yu, Takahara, & Prentice, 2001; Qiu et al., 2011; Tang et al., 2018), the relative roles of spatial arrangement (IBD), local adaptation (IBE), and historical climate fluctuations (IBI) in shaping spatial genetic patterns in East Asia have rarely been thoroughly examined (but see He, Wang, Li, & Yi, 2016; Zhang, Wang, Comes, Peng, & Qiu, 2016).

Multiple factors can potentially influence the relative roles of IBD, IBE, and IBI in shaping spatial genetic patterns, including the genetic markers, regions, or species examined. Phylogeographic studies traditionally use chloroplast and nuclear markers, which have differing modes of inheritance, mutation rate, ploidy, and effective population size. Hence, independent spatial genetic patterns can be characterized for the two sets of markers under the same species demographic history and have frequently been reported in phylogeographic studies [e.g., *Vriesea gigantea* Gaudich. (Palma‐Silva et al., 2009), *Arabidopsis halleri* (L.) O'Kane & Al‐Shehbaz (Pauwels et al., 2012), *Juglans mandshurica* Maxim. (Bai, Liao, & Zhang, 2010)]. Nonetheless, the examination of IBD‐, IBE‐, and IBI‐related patterns in nuclear markers tends to be considerably more frequent than that using chloroplast markers. We assume that this disparity in usage can be attributed to the fact that IBD, IBE, and IBI have been studied to a greater extent in landscape genetics (in which chloroplast markers tend to be assessed less frequently) than in phylogeographic (Manel, Schwartz, Luikart, & Taberlet, 2003), which reflects the slight difference between the two fields (Rissler, 2016; Wang, 2010). Despite the disparate status of these markers, we maintain that comparisons among IBD, IBE, and IBI patterns based on both markers types would be necessary for gaining a more comprehensive understanding of the processes occurring in different genomes in the same cell, which in turn can contribute disentangling the complex processes associated with the origins of biodiversity.

As its designation indicates, the SEA is located in a lower latitude region than the NEA, and is also characterized by a higher degree of physiographical heterogeneity. Both pollen records and genetic data indicate that the SEA region has had the potential to sustain more refugia for temperate flora than the NEA region during glacial periods (Qian & Ricklefs, 2004; Qiu et al., 2011). Hence, we presume that IBD, IBE, and IBI processes would have differed to certain extents in the NEA and SEA regions. Although some independent studies have examined these processes in the SEA or entire region (He et al., 2016; Zhang et al., 2016), the regional differences between the NEA and SEA regions have yet to be compared. Moreover, even within the same region, different species with differing traits may show contrasting responses to IBD, IBE, and IBI. Accordingly, from the perspective of assessing the relative roles of these different processes, studies of widespread species with sister clades in the NEA and SEA regions would contribute to minimizing species difference and maximizing regional differences.

*Lindera obtusiloba* Blume is a drought‐sensitive deciduous shrub or small tree that has a widespread distribution in both the NEA and SEA regions (Figure 1). In a previous study on this species (Ye et al., 2017), both chloroplast DNA sequence (cpDNA) and nuclear microsatellite (nSSR) data indicated the occurrence of two clearly discontinuous genetic clades in the NEA and SEA regions (Figure 1). Over the entire distribution range of *L. obtusiloba,* the greatest divergence in the populations of this species occurs between the NEA and SEA populations. Both the most recent common ancestor of cpDNA haplotypes and population divergence estimates based on nSSRs have dated the divergence between NEA and SEA populations to the Pliocene (5.33–2.58 Ma; Ye et al., 2017). Notable in this regard is the fact that the genetic break between NEA and SEA populations of *L. obtusiloba* is considerably sharper than that reported for other species with a similar distribution, for example, Asian butternuts (Bai et al., 2016), *Acer mono* Maxim. (Guo et al., 2014), *Kalopanax septemlobus* Koidz. (Sakaguchi et al., 2012), and *Prinsepia* Royle (Ma, Wang, Tian, & Sun, 2019). We suspect that this abrupt genetic break between *L. obtusiloba* populations could be attributed to the drought sensitivity of the species and that the arid belt located between NEA and SEA regions may represent a strong climatic barrier to the spread of *L. obtusiloba*.

The findings of both field and common garden studies have indicated that the NEA and SEA populations of *L. obtusiloba* can be distinguished with respect to seed weight and multiple phenological traits, including bud break and fruit maturation (Dai, 2019). In spite of such trait differences, however, the two genetic clades are sister clades that are characterized by similar climatic niches (Tian 2019, unpublished data). Hence, *L. obtusiloba* would appear to provide an ideal system for comparative analysis of evolutionary processes occurring in the NEA and SEA regions. In this regard, although Ye et al. (2017) have previously provided a detailed description of the spatial genetic patterns of *L. obtusiloba* in the NEA and SEA regions, there has to date been no comparative examination of the relative contributions of IBD, IBE, and IBI to present‐day distributions. Moreover, Ye et al. (2017) found that compared with SEA populations of *L. obtusiloba,* those in the NEA harbors a higher genetic diversity of nSSRs, although not of cpDNA, thereby implying the differential history of the two marker types. Accordingly, on the basis of a combination of cpDNA and nSSR data and ecological niche modeling (ENM), we examined IBD, IBE, and IBI patterns in both types of markers with respect to the overall range of *L. obtusiloba* and also that in the two divided subregions (NEA and SEA). Our goal was to identify potential differences in the evolutionary processes occurring in the different regions and for different types of genetic markers.

## 2 METHODS

### 2.1 Genetic differentiation

In the present study, we used a data set previously compiled by Ye et al. (2017), which comprises the genotypic data of six nSSRs for 659 individuals and those of four cpDNA fragments (*psbA*–*trnH*, *trnL*–*trnF*, *trnS*–*trnG*, and *rpl16*) for 271 individuals. The individuals analyzed were derived from 28 populations (10 in the NEA and 18 in the SEA; Figure 1; Appendix S1). The sampled populations were scattered across an area representing the major distribution of the species. More than three voucher specimens of each sampled population have been archived in the herbarium of Beijing Normal University. The cpDNA data have been deposited in GenBank with the accession nos. KU645591–KU645758. Multiple population genetic distance indices were determined for both nSSRs and cpDNA, which constitute a comprehensive map of population genetic divergence (Appendix S2). For cpDNA, we calculated the population genetic distance indices *N_ST_*, *G_ST_*, and *D_xy_* using DnaSP version 6.12.03 (Rozas et al., 2017), and for nSSRs, the population genetic distance indices *F_ST_* and *R_ST_* were calculated using GenAlEx 6.5 (Peakall & Smouse, 2012) and SPAGeDi 1.5 (Hardy & Vekemans, 2002), respectively. All calculations for genetic distance indices were performed using the default settings in each program.

### 2.2 Geographical distance

We represented the locations of the sampled populations in terms of longitude and latitude under the Beijing 1954 projection system. Pairwise geographical distances between sampled populations were calculated by using the function “distm” in the package “geosphere” (Hijmans, 2015) of R version 3.6.0 (R Development Core Team, 2013). For distance computation, we applied the “distVincentyEllipsoid” function, which calculates the shortest circle distance between points.

### 2.3 Environmental distance

As environmental variables, we used the data of 19 bioclimatic variables for the current period (1950–2000) obtained from the WorldClim database (Fick & Hijmans, 2017; Hijmans, Cameron, Parra, Jones, & Jarvis, 2005; Appendix S3). All climate layers used in this study were prepared at a resolution of 2.5 arc minutes. For each of the 28 sampling sites, the 19 bioclimatic variables were extracted using ArcGIS 10.1 (ESRI). To characterize major environmental variation, we performed principal component analysis (PCA) using the “prcomp” function in R version 3.6.0 (R Development Core Team, 2013). Given that the first two coordinates of PCA (PC1 and PC2) can account for most of the environmental variability, we calculated the environmental distances between each of the sampled populations on the first two PC axes using the “vegdist” function based on the Euclidean method in the “vegan” package (Oksanen, Blanchet, Kindt, Legendre, & Minchin, 2017) of R version 3.6.0.

### 2.4 Resistance distance

To assess the influence of the historical instability of habitat connectivity on population genetic differentiation, we modeled the distributions of suitable habitats for the present, last glacial maximum (LGM; 21 thousand years ago, hereafter, kya), and last interglacial (LIG; 120 kya) periods based on ecological niche modeling (ENM) in MAXENT (Phillips & Dudík, 2008). The ENM procedure and the results obtained using this procedure have been described in detail in our previous study (Ye et al., 2017). In brief, 100 occurrence sites of *L. obtusiloba* showing no spatial redundancy were included in the ENM. The locations of these 100 sites and data sources can be found in Appendix S4. The modeling included only 11 bioclimatic variables that lacked strong correlation (*r* < .9), namely annual mean temperature, mean diurnal range, isothermality, temperature seasonality, maximum temperature of the warmest month, minimum temperature of the coldest month, mean temperature of the wettest quarter, annual precipitation, precipitation of the wettest month, precipitation of the driest month, and precipitation seasonality. Model validation was performed via 10 independent runs with 20% randomly selected data. High receiver operating characteristic (ROC) values (0.928 ± 0.025) have been reported for this type of modeling, which indicate its good accuracy. The established model was projected onto LGM (MIROC‐ESM) and LIG climatic models (Shields et al., 2012; Watanabe et al., 2011).

**FIGURE 2:**
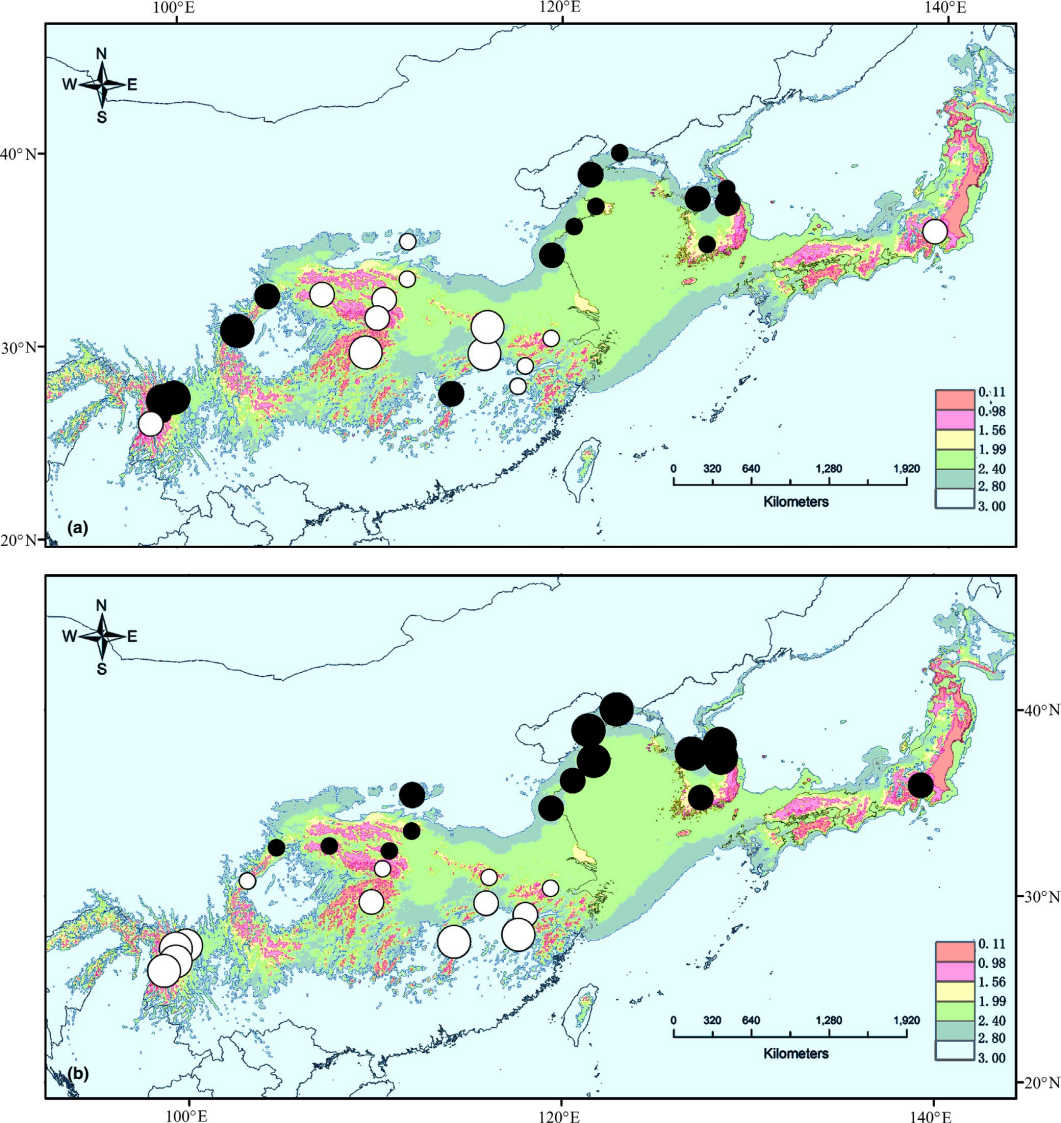
The geographical distribution of population genetic distances for (a) the nSSR index *R_ST_* and (b) the cpDNA index *D_XY_*. The population genetic distance of each sampled populations is represented by the average *R_ST_* (or *D_XY_*) with that of each of the other populations. A higher than the species‐level average *R_ST_* (or *D_XY_*) value is represented by a black circle, whereas a lower than average value is denoted by a while circle. The size of the circles is proportional to the difference from the average value. The background map in both (a) and (b) is the historical climatic instability resistance map, with 0 representing the lowest climate instability resistance due to the highest habitat suitability in each period [last interglacial (LIG), last glacial maximum (LGM), and the present), and 3 representing the highest climate instability resistance due to the lowest habitat suitability in each period

The climatic suitability of each grid in each period was described in terms of the species occurrence possibility obtained from the ENM results for *L. obtusiloba*. To calculate resistance distance, the climatic suitability of each period was converted into a resistance value as climatic unsuitability (“1‐suitability”), using the “create friction layer” function in the SDM toolbox (Brown, 2014; Brown, Bennett, & French, 2017). The historical climate instability resistance map was represented by the overall climate unsuitability from LIG to the present, calculated by adding the unsuitability values of each period. The values of the resistance map ranged from 0 to 3, with 0 representing the lowest climate instability resistance due to highest habitat suitability in each period and 3 representing the highest climate instability resistance due to lowest habitat suitability in each period (Figure 2).

On the basis of the historical climate instability resistance map, we calculated resistance distance among 28 populations according to circuit theory in Circuitscape v4.0 (McRae, Dickson, Keitt, & Shah, 2008; Shah & McRae, 2008).

### 2.5 IBD, IBE, and IBI analyses

To investigate the relationships between ecological factors (geographical, environmental, and historical habitat instability) and population genetic differentiation, we performed Mantel tests with 10,000 permutations for each genetic distance index of both marker types (nSSRs: *F_ST_* and *R_ST_*; cpDNA: *N_ST_*, *G_ST_*,and *D_xy_*). To disentangle the potentially complex interactions among geographical distance, environmental distance, and resistance distance and compare the relative contribution of IBD, IBE, and IBI on population differentiation, we used multiple matrix regression with randomization (MMRR) based on 10,000 permutations. All variables included in the aforementioned analyses were standardized using the “scale” function in the “vegan” package (Oksanen et al., 2017). Both the Mantel test and MMRR analysis were performed using the R program (R Development Core Team, 2013).

To compare the processes shaping the spatial genetic structure in the NEA and SEA regions, we performed the same Mantel test and MMRR analyses using only data pertaining to the NEA or SEA, respectively.

When performing analyses, we did not take into consideration nonlinear relationships. Although certain variables probably showed nonlinear relationships with genetic distance, their linear relationships were still significant in this study. Moreover, whereas a linear fit may not the best fit for these relationships, from the perspective of simplification, we only included linear relationships for variables when comparing the relative contributions of IBD, IBE, and IBI to spatial genetic patterns. Therefore, the use of a linear model may represent a more conservative test of the relationships.

## 3 RESULTS

### 3.1 Environmental analyses

The results of PCA indicated that the first two coordinates (PC1 and PC2) accounted for 67.24% of the environmental variation. For PC1, environmental variables related to humidity (e.g., annual precipitation, precipitation of the wettest quarter, precipitation of the driest quarter, and precipitation of the coldest quarter) had the highest weights, whereas for PC2, temperature‐related variables (e.g., isothermality, temperature seasonality, maximum temperature of the warmest month, and temperature annual range) had the highest weights (Appendix S3). Neither PC1 nor PC2 values showed significant differences between the NEA and SEA regions, whereas the points pertaining to the NEA populations were clustered when combined PC1 and PC2, implying that habitat in the NEA was probably a marginal type of that in the SEA (Figure 1c).

ENM results (Ye et al., 2017) indicated that the area of habitats in the SEA with suitable climates was larger during the LGM than the present‐day distributions, whereas the area in the NEA was more climatically limited. Furthermore, during the LIG, the area of suitable habitats in both NEA and SEA regions was smaller than that during the present and LGM periods.

Climate instability resistance values, which were obtained based converting the ENM results and summing over the three periods, showed an uneven spatial distribution (Figure 2). We found that the central SEA region and the Japanese Archipelago in the NEA had larger continuous ranges of long‐term stable habitats than the remaining areas of species' range, which indicates a lower climate instability resistance for long‐term gene flow in these two regions. Interestingly, the population genetic differentiation of nSSRs was found to be generally lower in the central SEA region and Japanese Archipelago (Figure 2a), whereas no comparable pattern was observed with respect to cpDNA (Figure 2b).

### 3.2 IBD, IBE, and IBI in nSSRs

**FIGURE 3:**
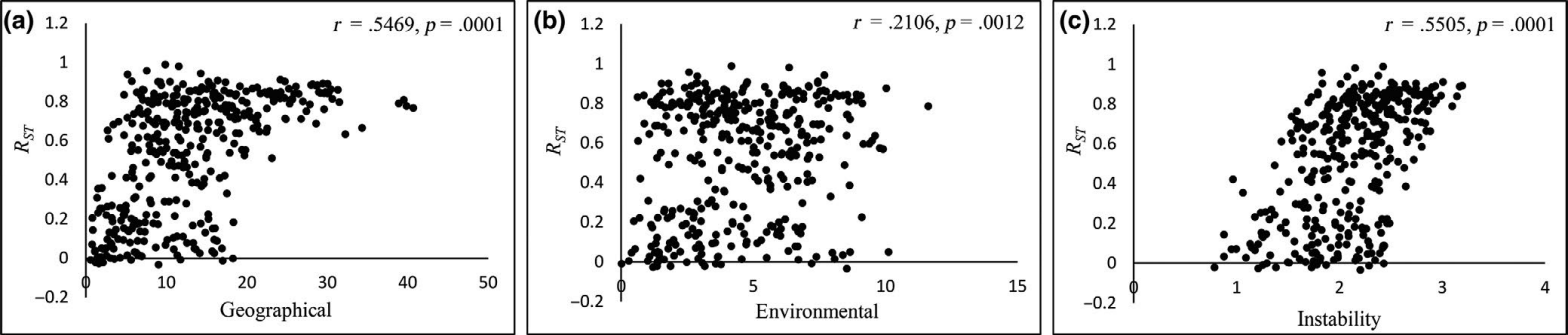
Scatter plots showing the relationships between the population genetic distance index *R_ST_* (nSSR) and (a) geographical distance, (b) environmental distance, and (c) climatic instability resistance distance. The statistical results of Mantel tests are shown in the upper right‐hand corner of each plot

nSSR analysis indicated that in both the NEA and SEA regions, *F_ST_* and *R_ST_* showed significantly positive relationships with geographical distance (*F_ST_*: *r* = .5221, *p* = .0001; *R_ST_*: *r* = .5469, *p* = .0001) and climatic instability resistance (*F_ST_*: *r* = .5781, *p* = .0001; *R_ST_*: *r* = .5505, *p* = .0001) based on the Mantel test (Figure 3; Appendix S5). MMRR analysis indicated that climatic instability resistance (*F_ST_*: β = 0.1230, *p* = .0001; *R_ST_*: β = 0.1308, *p* = .0001) can explain more variance in *F_ST_* or *R_ST_* than geographical distance (*F_ST_*: β = 0.0577, *p* = .0103; *R_ST_*: β = 0.1035, *p* = .0003; Table 1), thereby indicating a stronger IBI pattern than that of IBD. In the MMRR analysis (Table 1), *F_ST_* showed a significant negative relationship with environmental distance (β = −0.0468, *p* = .0024), whereas the Mantel test indicated that *R_ST_* has a significantly positive relationship with environmental distance (*r* = .2106, *p* = .0012). This seemingly contradictory pattern may indicate a weak effect of IBE over the entire species range.

**TABLE 1:** Multiple matrix regression with randomization (MMRR) analysis results showing the relative contribution of isolation by spatial distance (IBD), environment (IBE), and historical climatic instability (IBI) with respect to multiple population genetic distance indices of both nSSRs and cpDNA markers in the overall range (overall) of *Lindera obtusiloba* and two subregions (NEA and SEA) within this range

Regions	Genetic divergence	β_Total_	β_IBD_	β_IBE_	β_IBI_
Overall nSSRs	*F_ST_*	0.6227**	0.0577*	−0.0468**	0.123**
	*R_ST_*	0.5898**	0.1035**	−0.0261	0.1308**
Overall cpDNA	*G_ST_*	0.4056*	−0.1445*	0.0932*	0.0924
	*N_ST_*	0.3746**	0.0169	0.0791**	0.0178
	*D_xy_*	0.4211**	0.0006**	−0.0001	0.0002
NEA nSSRs	*F_ST_*	0.6503*	0.0229	−0.0002	0.0525
	*R_ST_*	0.6101*	−0.0794**	−0.0065	0.2067**
NEA cpDNA	*G_ST_*	0.454	−0.1195	0.0316	0.2689
	*N_ST_*	0.4534	−0.1369	0.0344	0.3404
	*D_xy_*	0.4464	0.0002	−0.0001	0.0000
SEA nSSRs	*F_ST_*	0.4064*	−0.0118	−0.0443*	0.1153*
	*R_ST_*	0.4710**	0.0126	−0.0844*	0.2226**
SEA cpDNA	*G_ST_*	0.3587	−0.1422	0.0393	0.1706
	*N_ST_*	0.4062*	0.0411	0.0282	0.0546
	*D_xy_*	0.5961**	0.0010**	−0.0001	−0.0005

* .01 < *p* < .05;

** *p* < .01.

Both the Mantel test and MMRR analysis indicated a significantly positive relationship between climatic instability resistance and *F_ST_*/*R_ST_* (Mantel: *F_ST_*: *r* = .3279, *p* = .0064; *R_ST_*: *r* = .4237, *p* = .0004; MMRR: *F_ST_*: β = 0.1153, *p* = .0393; *R_ST_*: β = 0.2226, *p* = .0017; Table 1; Appendix S7), which is indicative of a strong IBI pattern. Geographical distance showed a significantly positive relationship with *R_ST_* based on the Mantel test, whereas we were unable to detect any significant relationships when performing MMRR analysis. In contrast, environmental distance showed a significant negative relationship with both *F_ST_* (β = −0.0443, *p* = .0402) and *R_ST_* (β = −0.0844, *p* = .0227) based on the MMRR analysis, whereas significant relationships were detected using the Mantel test. The contrasting patterns for geographical and environmental distances identified using the Mantel test and MMRR analysis may indicate the weak effects of these two factors on the nuclear genetic patterns in the SEA region.

For the NEA region, the weak IBI pattern we identified may be attributable to the fact that only *R_ST_* showed a relatively consistent IBI pattern. Although the Mantel test did not identify significant relationships for *R_ST_*, with respect to geographical and environmental distances, it indicates a marginally significant positive relationship with climatic instability resistance (*r* = .2657; *p* = .0784; Appendix S8), whereas MMRR analysis identified a positive relationship with climatic instability resistance (β = 0.2067; *p* = .0071) and a negative relationship with geographical distance (β = −0.0794; *p* = .0021; Table 1). With respect to *F_ST_*, the Mantel test indicated a significant positive relationship with both geographical distance (*r* = .5920, *p* = .0135) and climatic instability resistance (*r* = .5817, *p* = .0003), whereas MMRR analysis failed to identify any significant relationships for the three distance indices. Thus, on the basis of the present analyses, we were unable to identify any clear patterns in IBD or IBE in the NEA region.

### 3.3 IBD, IBE, and IBI in cpDNA

**FIGURE 4:**
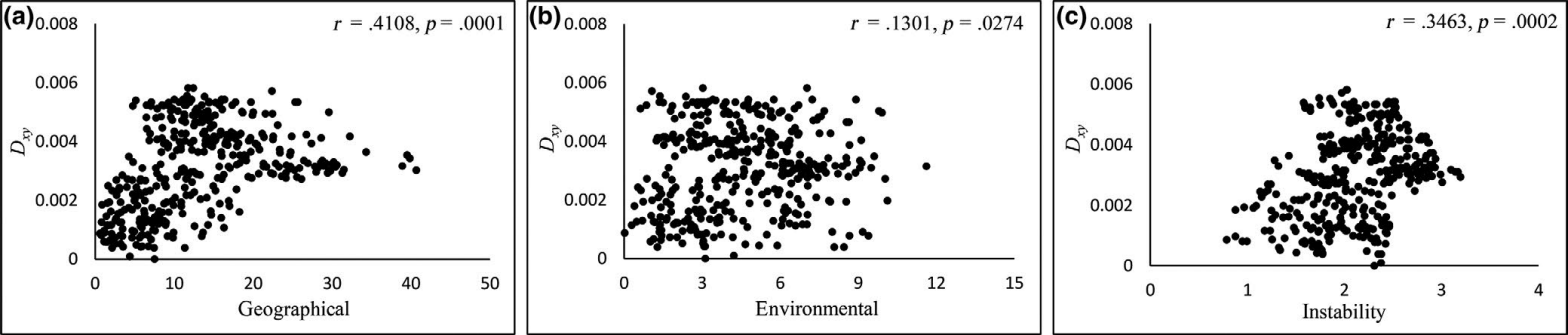
Scatter plots showing the relationships between the population genetic distance index *D_XY_* (cpDNA) and (a) geographical distance, (b) environmental distance, and (c) climatic instability resistance distance. The statistical results of Mantel tests are shown in the upper right‐hand corner of each plot

Our analyses of the chloroplast genome revealed differences in the correlation patterns of the three genetic distance indices throughout the entire distribution range of *L. obtusiloba*. Using the Mantel test, we found that genetic distance *D_xy_*, which indicates divergence since the last common ancestor between populations, was significantly correlated with the three factors (Figure 4), with the highest correlation value observed for geographical distance (*r* = .4108, *p* = .0001). MMRR analysis revealed that *D_xy_* was only influenced by geographical distance (β = 0.0006, *p* = .0007; Table 1). Genetic distance *N_ST_* takes into account both the allele frequency between populations and allele genetic distance, and was found to be significantly correlated with the three factors in the Mantel test, with the highest correlation value (*r* = .3607, *p* = .0001) being identified for environmental distance (Appendix S6), whereas *N_ST_* was only influenced by environmental distance based on the MMRR analysis (β = 0.0791, *r* = .0008; Table 1). The disparity between the results obtained using the Mantel test and MMRR analysis may be attributable to the collinearity between geographical distance and climatic instability resistance (β = 0.758, *p* = .000). In this regard, we suspect that the results of MMRR analysis may be more accurate than those obtained the Mantel test, as MMRR simultaneously considers all factors. For *G_ST_*, which only applies to the allele frequency between populations, both the Mantel test and MMRR analysis showed significantly positive correlation with environmental distance (Mantel test: *r* = .2025, *p* = .0381; MMRR analysis: β = 0.0932, *r* = .0146; Table 1; Appendix S6). *G_ST_* appeared to show no clear IBD pattern, given that the Mantel test revealed no significant correlations and MMRR analysis revealed an anomalously significant negative relationship.

For the SEA region, we found that the distribution of *D_xy_* shows a pattern similar to that observed for the entire species range, with a significant IBD pattern (Mantel test: *r* = .5459, *p* = .0001; MMRR analysis: β = 0.0010, *p* = .0003) but no clear IBE or IBI patterns (Appendix S7). Although MMRR analysis indicated that neither *N_ST_* nor *G_ST_* showed significant IBD, IBE, or IBI patterns (Table 1), the Mantel test indicated that *N_ST_* showed a significantly positive correlation with all three factors when tested independently (IBD, *r* = .3752, *p* = .0001; IBE, *r* = .3344, *p* = .0002; IBI, *r* = .3690, *p* = .0027; Appendix S7).

With the exception of a marginally significant positive correlation between *D_xy_* and geographical distance (Mantel test: *r* = .4112, *p* = .0670; MMRR analysis: β = 0.0002, *p* = .0926), we were unable to detect any significant IBD, IBE, or IBI patterns with respect to genetic distance indices in the NEA region (Table 1; Appendix S8).

## 4 DISCUSSION

### 4.1 Different processes underlying genetic differentiation in the nuclear and chloroplast genome

In this study, we identified differences in the processes underlying the divergent genetic patterns in nSSRs and cpDNA in *L. obtusiloba*. With respect to nSSRs, we detected significant IBI and IBD patterns over the entire species range, with the contribution of IBI being found to be greater than that of IBD. Given that neither of the NEA and SEA subregions show a clear IBD pattern, we infer that the observed IBD pattern over the entire range may have been primarily influenced by the greatest population divergence between the NEA and SEA regions, which has been reported previously by Ye et al. (2017). Thus, climatic instability since the late Pleistocene is likely to have been the main driving force underlying the nuclear genetic patterns observed in *L. obtusiloba*.

Our observations indicate that climate instability resistance between populations would not appear to explain the genetic divergence of cpDNA. However, the genetic distance index *D_xy_* shows a clear IBD pattern, and the genetic differentiation of the indices *N_ST_* and *G_ST_* shows a clear IBE pattern over the entire range of the species. We suspect that local adaptation to different environments in the NEA and SEA regions (Figure 2) may have driven chloroplast divergence in the population. Generally, the NEA region is considered to have greater seasonality than the SEA region with respect to temperature and precipitation (Bai et al., 2016; Qiu et al., 2011). Although we were unable to detect any significant differences between the habitats of *L. obtusiloba* in NEA and SEA regions in relation to humidity (PC1) nor heat (PC2), the habitat types in the NEA are unique when these two climatic factors are considered together. We suspect that habitat in the NEA region is a marginal type of that in the SEA region (Figure 1c; Appendix S4). Local adaptation in marginal habitats has been reported in several species, including *Arabidopsis lyrata* (Linnaeus) O'Kane & Al‐Shehbaz (Hämälä, Mattila, & Savolainen, 2018), *Ambrosia artemisiifolia* L. (van Boheemen, Atwater, & Hodgins, 2019), and *Mimulus guttatus* DC. (Ferris, Barnett, Blackman, & Willis, 2017). In addition, population divergence of *L. obtusiloba* in the two regions is estimated to have undergone divergence in the Pliocene and gene flow between the two regions is rare (Ye et al., 2017). Hence, local adaptations may have evolved in the two regions, and indeed, differences of multiple functional traits between populations in the NEA and SEA regions, including fruit morphology and the phenology of bud break and fruit maturation, have been observed in either field or common garden studies (Dai, 2019). Compared with IBD or IBI, the IBE patterns detected in the genome can be heterogeneous, as these patterns tend to be driven primarily by selection against immigration (Sexton et al., 2014; Wang & Bradburd, 2014). Therefore, analysis of a limited number of nSSR loci may not be sufficient to detect a significant IBE pattern in the nuclear genome.

The heredity of nuclear and chloroplast genomes may have contributed to the contrasting responses of these genomes to historical climate instability. The chloroplasts in our study species are maternally inherited and can only be transmitted to offspring by seed flow, whereas the nuclear genomes are biparentally inherited and can be transmitted via both seed and pollen flows. Dispersal of the seeds and pollen of *L. obtusiloba* is mediated by birds and insects, respectively (Smith, Hamel, Devall, & Nathan, 2004), and previous phylogeographic studies and fossils data indicate that climate change can affect the distribution of these vector species (Dong et al., 2017; Kharouba, Lewthwaite, Guralnick, Kerr, & Vellend, 2019; Wang, Zhu, Heller, Zhou, & Shi, 2020). Hence, it is to be assumed that dispersal based on these species would be influenced to a considerable extent by climate fluctuations (Guisan & Thuiller, 2005; Pearson & Dawson, 2003; Van der Putten, Macel, & Visser, 2010). Moreover, compared with birds, insects typically have a limited range of dispersal (Phillipsen et al., 2015; Phillipsen & Lytle, 2013). Accordingly, given that patterns in the nuclear genome can reflect the dispersal patterns of both seed and pollen, the effect of climate instability on bird and insect distribution may have resulted in a stronger IBI pattern in the nuclear genome compared with that of the chloroplast.

In addition to the contrasting modes of heredity, differences in ploidy may also have contributed to the different correlation patterns we detected for the two genomes. The nuclear genome is diploid, undergoes recombination, and generally has a larger effective population size than the chloroplast, which is haploid and does not recombine (Gaudeul, Gardner, Thomas, Ennos, & Hollingsworth, 2014; McCauley, 1994). Hence, the nuclear genome might be excepted to have a higher mutation rate and genetic diversity than the chloroplast, which could reflect the greater effect the more substantial influence of recent historical climate change on the gene flow between populations (Bai et al., 2010). In addition, nSSRs, which are markers commonly used in phylogeographic studies, have a considerably higher mutation rate than cpDNA sequences, which may enhance the observed IBI pattern. In summary, differences in heredity and ploidy between the nuclear and chloroplast genomes or the differences between nSSR and cpDNA sequences could account for the stronger IBI pattern in nSSRs that we observed in the present study.

### 4.2 Genetic patterns in the SEA region are more pronounced than those in the NEA region

Although we identified similarities in the correlation patterns detected for the NEA and SEA regions, we found the patterns in the SEA region to be considerably more significant than those in the NEA region. With respect to nSSRs, both *F*_ST_ and *R*_ST_ showed a significant IBI pattern in the SEA region, whereas only *R_ST_* showed a significant IBI pattern in the NEA region. For cpDNA, whereas *D_xy_* showed a significant IBD pattern in the SEA region, the pattern for this index was only marginally significant in the NEA region. We suspect that the stronger IBI and IBD patterns detected for SEA region could be related to a combination of topographical differences and Pleistocene climatic oscillations.

Populations of temperate plants in the NEA and SEA regions probably underwent different rates and extents of expansion and contractions in response to Pleistocene climate fluctuations (Milne & Abbott, 2002; Qiu et al., 2011). In the SEA region, both molecular and ENM evidence indicate that numerous locations in the western, central, and eastern mountainous areas may have served as glacial refugia for *L. obtsuloba* (Ye et al., 2017). The combination of climate instability and geological isolations would thus have led to a negative correlation between gene flow and resistance or geographical distance, thus resulting in significant IBI or IBD patterns.

Although molecular data for the NEA region indicate long‐term persistence, most populations, particularly those on the mainland, were formed recently via postglacial migration from the southern margins of the NEA or Japanese Archipelago. Inferences with respect to NEA and SEA glacial refugia are also supported by the distribution of climatically stable habitats during the late Pleistocene (Figure 2). These findings indicate that most NEA populations have a more recent population history than those in the SEA region. In addition, the southern clades in the SEA region have a considerably wider distribution than clades in the NEA region. Although the number of populations we sampled in the NEA (10 populations) was smaller than the number examined in the SEA region (18 populations), we do not believe this to be a prominent factor accounting for the less pronounce genetic patterns detected in the NEA. Relative to their respective range sizes, sampling densities in the two regions were similar, and thus, the influence of sampling effort on characterizing spatial variance in the two regions would be comparable. Insufficient time and/or space can restrict the effects of evolutionary forces (such as drift or selection) and thereby reduce the significance of detected patterns (Holderegger, Buehler, Gugerli, & Manel, 2010; Sexton et al., 2014). In the NEA region, the shorter evolutionary history and relatively homogeneous topographical have probably been important factors contributing to limited drift and geographical isolation. Moreover, postglacial expansion occurred in a restricted population range in the mainland area of the NEA region, which may also have contributed to limited population genetic divergence. Hence, the less prominent IBI or IBD patterns we identified in the NEA region may be related to the recent population history and restricted population range of *L. obtusiloba* in this region.

## 5 CONCLUSIONS

In this study, we compared the relative contributions of IBD, IBE, and IBI to the genetic divergence of *L. obtusiloba* in East Asia, based on analyses of different types of genetic marker and different regions. We found that different processes have probably driven the patterns of nuclear and chloroplast genetic divergence. The divergence of nSSRs appears to have been influenced primarily by the historical climatic instability (IBI) over either the entire species range or within the two distinct subregions (NEA and SEA) of this range. In contrast, we were unable to detect any significant IBI pattern with respect to the divergence of cpDNA. We envisage that changes in climatic change during the late Pleistocene have had a stronger influence on population divergence of the nuclear genome than that of the chloroplast genome, which can probably be attributed to differences in the inheritance mode and ploidy of these two genomes. Differences in the patterns of cpDNA divergence are assumed to be influenced by spatial distribution (IBD). In addition, local adaptation may have affected the divergence of the chloroplast between the northern and southern regions, whereas we detected no clear IBE pattern for nuclear divergence based on the set of nuclear loci analyzed in the present study.

We also identified convergence in the correlation patterns of both the nuclear and chloroplast genomes in the NEA and SEA regions. However, due to the shorter population history and more restricted population range of *L. obtusiloba* in the NEA, we found this region to be characterized by a less significant pattern of genetic divergence than the SEA region. Collectively, the findings of this study serve to emphasize that even within a single species, analysis of different genetic markers or different regions can reveal differences in IBD, IBE, or IBI patterns or differing extent of significance. Such findings will contribute to gaining a more complete understanding of the population history of different genomes or patterns of divergence in different regions of a species' range.

## Supporting information

Appendix S1

Appendix S2

Appendix S3

Appendix S4

Appendix S5‐S8

## Data Availability

The chloroplast DNA sequence data have been deposited in GenBank with accession nos. KU645591–KU645758. Data relating to MaxEnt files, and microsatellite genotypes have been uploaded to DRYAD: https://datadryad.org/stash/share/4ihCPpUOWD_zNdlbsTdxDwWD5uGcM7Qm0sI6LcW-5aE.
