# Supplementary material for: Different processes shape the patterns of divergence in the nuclear and chloroplast genomes of a relict tree species in East Asia
Source: Ecol Evol. 2020 Mar 24;10(10):4331–42. doi: 10.1002/ece3.6200 (PMC7246201; doi:10.1002/ece3.6200)
Supplement: Supplementary file 2 — Appendix S2 [file ECE3-10-4331-s002.docx]

**Appendix 2** Different population genetic distance indices in nSSRs and cpDNA calculated in this study

| Parameters | Equation | Parameters | Impacted Factors |
| --- | --- | --- | --- |
| *F_ST_* | $F_{ST}=\frac{\sigma_{S}^{2}}{\sigma_{T}^{2}}$ | $\sigma_{S}^{2}$ means allele frequencies between subpopulations; $\sigma_{T}^{2}$ means allele frequencies in total population. | Many evolutionary forces could affect *F_ST_* , with increased by genetic drift and reduced by migration (Nei and Kumar 2000, Balloux and Lugon-Moulin 2002, Hedrick 2005, Meirmans and Hedrick 2011). |
| *R_ST_* | $R_{ST}=\frac{S-S_{w}}{S}$ | S means squared difference in allele size for genes in same population; S_w_ means squared difference in allele size for different populations. | *R_ST_* takes into account allele size, provide more information on population differentiation. If populations have exchanges migrants or diverged for long time, *R_ST_* values are expected lower (Balloux and Lugon-Moulin 2002, Hardy et al. 2003). |
| *G_ST_* | $G_{ST}=\frac{H_{T}-H_{S}}{H_{T}}$ | H_T_ means the total diversity of population; H_S_ means average between subpopulation diversity. | *G_ST_* has straightforward with gene flow and mutation rate, such as genetic drift and migration (Pons and Chaouche 1995, Hedrick 2005, Meirmans and Hedrick 2011). |
| *N_ST_* | $N_{ST}=\frac{\pi_{T}-\pi_{S}}{\pi_{T}}$ | $\pi_{T}$ means nucleotide diversity for all populations; $\pi_{S}$ means nucleotide diversity within populations. | *N_ST_* can be account for genetic distance and allele frequences of nucleotide differentiation. Compared *N_ST_* with *G_ST_* could be insight into present species subdivision (Pons and Petit 1996, Nei and Kumar 2000). |
| *D_xy_* | $D_{xy}=\sum_{ij} x_{i}y_{i}d_{ij}$ | *d_ij_* measures the number of nucleotide differences between the *i^th^* the *j^th^* haplotype from population X and Y | Interpopulation diversity was not affected *D_xy_*, compared with ancestral levels of diversity and substitution rates (Nozawa and Shotake 1990, Petersen et al. 1994, Cruickshank and Hahn 2014). |
