# Supplementary material for: Different processes shape the patterns of divergence in the nuclear and chloroplast genomes of a relict tree species in East Asia
Source: Ecol Evol. 2020 Mar 24;10(10):4331–42. doi: 10.1002/ece3.6200 (PMC7246201; doi:10.1002/ece3.6200)
Supplement: Supplementary file 3 — Appendix S3 [file ECE3-10-4331-s003.docx]

**Appendix 3** Bioclimatic variables, standardized loadings for the first two axes of the principle component analysis (PCA).

| Variable | Description | PC1 | PC2 |
| --- | --- | --- | --- |
| bio1 | Annual mean temperature | 0.252 | 0.000 |
| bio2 | Mean diurnal range (mean of monthly (max temp - min temp)) | 0.000 | -0.183 |
| **bio3** | **Isothermality (BIO2/BIO7) (× 100)** | 0.000 | **-0.379** |
| **bio4** | **Temperature seasonality (SD × 100)** | -0.176 | **0.381** |
| **bio5** | **Max temperature of warmest month** | 0.000 | **0.347** |
| bio6 | Min temperature of coldest month | 0.276 | -0.154 |
| **bio7** | **Temperature Annual Range (BIO5-BIO6)** | -0.194 | **0.341** |
| bio8 | Mean temperature of wettest quarter | 0.000 | 0.257 |
| bio9 | Mean temperature of driest quarter | 0.287 | -0.182 |
| bio10 | Mean temperature of warmest quarter | 0.000 | 0.335 |
| bio11 | Mean temperature of coldest quarter | 0.265 | -0.214 |
| **bio12** | **Annual precipitation** | **0.332** | 0.000 |
| bio13 | Precipitation of wettest month | 0.233 | 0.000 |
| bio14 | Precipitation of driest month | 0.268 | 0.241 |
| bio15 | Precipitation seasonality (coefficient of variation) | -0.246 | -0.102 |
| **bio16** | **Precipitation of wettest quarter** | **0.288** | 0.000 |
| **bio17** | **Precipitation of driest quarter** | **0.292** | 0.208 |
| bio18 | Precipitation of warmest quarter | 0.254 | 0.000 |
| **bio19** | **Precipitation of coldest quarter** | **0.298** | 0.188 |
