# Supplementary material for: Different processes shape the patterns of divergence in the nuclear and chloroplast genomes of a relict tree species in East Asia
Source: Ecol Evol. 2020 Mar 24;10(10):4331–42. doi: 10.1002/ece3.6200 (PMC7246201; doi:10.1002/ece3.6200)
Supplement: Supplementary file 5 — Appendix S5‐S8 [file ECE3-10-4331-s005.docx]

**Appendix 5** Regression of pairwise nSSRs genetic differentiation (*F_ST_*) and geographic, environmental, climatic instability distance among all population. Red spots mean p < 0.05, while black spots mean p > 0.05, the same meaning to below.

**Appendix 6** Regression of pairwise cpDNA genetic differentiation (*G_ST_*) and geographic, environmental, climatic instability distance among all population, regression of pairwise cpDNA genetic differentiation (*N_ST_*) and geographic, environmental, climatic instability distance among all population.

**nSSRs pattern in SEA**

**cpDNA pattern in SEA**

**Appendix 7** Regression of pairwise genetic differentiation (*F_ST_*) and geographic, environmental, climatic instability distance among SEA population, regression of pairwise genetic differentiation (*R_ST_*) and geographic, environmental, climatic instability distance among SEA population, Regression of pairwise genetic differentiation (*G_ST_*) and geographic, environmental, climatic instability distance among SEA population, regression of pairwise cpDNA genetic differentiation (*N_ST_*) and geographic, environmental, climatic instability distance among SEA population, regression of pairwise cpDNA genetic differentiation (*D_xy_*) and geographic, environmental, climatic instability distance among SEA population.

**nSSRs pattern in NEA**

**cpDNA pattern in NEA**

**Appendix 8** Regression of pairwise genetic differentiation (*F_ST_*) and geographic, environmental, climatic instability distance among NEA population, regression of pairwise genetic differentiation (*R_ST_*) and geographic, environmental, climatic instability distance among NEA population, Regression of pairwise genetic differentiation (*G_ST_*) and geographic, environmental, climatic instability distance among NEA population, regression of pairwise cpDNA genetic differentiation (*N_ST_*) and geographic, environmental, climatic instability distance among NEA population, regression of pairwise cpDNA genetic differentiation (*D_xy_*) and geographic, environmental, climatic instability distance among NEA population.
